# P-859. Shorter versus Longer Duration of Antibiotic Therapy for Gram-Negative Bacteremia (GNB) in Immunocompromised Patients

**DOI:** 10.1093/ofid/ofae631.1051

**Published:** 2025-01-29

**Authors:** Nicole Slain, Olivia Randazza, Alex D Taylor, John C Williamson, Charles Hartis, Michael E DeWitt, Elizabeth Palavecino, Christopher Ohl, Vera P Luther, Ryan C Maves, Mary Banoub

**Affiliations:** University of Kentucky HealthCare, Lexington, Kentucky; Atrium Health Wake Forest Baptist, Winston-Salem, North Carolina; Atrium Health Wake Forest Baptist, Winston-Salem, North Carolina; Atrium Health Wake Forest Baptist, Winston-Salem, North Carolina; Atrium Health Wake Forest Baptist, Winston-Salem, North Carolina; Atrium Wake Forest Baptist Health/ Wake Forest University School of Medicine, Winston-Salem, North Carolina; Wake Forest School of Medicine, Winston Salem, North Carolina; Wake Forest School of Medicine, Winston Salem, North Carolina; Wake Forest University School of Medicine, Winston Salem, NC; Wake Forest University School of Medicine, Winston Salem, NC; Atrium Health Wake Forest Baptist Medical Center, Winston Salem, North Carolina

## Abstract

**Background:**

Although there is no standard duration of therapy for GNB, 14 days has been commonly used. In recent years, studies have evaluated shorter durations of antibiotics (abxs) specifically for uncomplicated GNB in immunocompetent patients. The purpose of this study was to determine whether a shorter duration of abx therapy for GNB in immunocompromised patients had an impact on clinical outcomes compared to a longer abx duration.

**Methods:**

This was a single-center, retrospective, matched pairs study from July 2021 to August 2023. Patients were included if they were ≥ 18 years old, hospitalized with GNB, and met immunocompromising criteria. A blood culture report was obtained and cross referenced with ICD-10 codes to identify patients. Short durations of abxs were considered ≤ 8 days, while a long duration was ≥ 10 days. The primary outcomes were all-cause mortality and abx failure at 90 days (defined as a relapsed blood stream infection, new local suppurative complication, or a new distant complication). Secondary outcomes consisted of 30 day infection related hospital readmission, length of stay, *Clostridioides difficile* infections, total active days of therapy, and development of resistance. Patients were matched in a 1:1 ratio based on type of GNB (*Enterobacterales* vs non-*Enterobacterales*), age (+/- 10 years) and Pitt Bacteremia Score (+/- 1).

**Results:**

29 matched pairs were included (Figure 1). The majority of matched pairs (96.6%) had *Enterobacterales* infections. The most common immunocompromising conditions were diabetes (21/29 and 19/29) and solid tumors (12/29 and 8/29) in the short and long groups, respectively. Most infections originated from the urinary tract, 16 (55.2%) in the short group and 20 (69.0%) in long group (Table 1). All-cause mortality within 90 days occurred in 2 (6.9%) patients in both the short and long groups (*P value* >0.9). Abx failure occurred in 1 (3.4%) patient in the short group and 2 (6.9%) in the long group (*P value* >0.9) (Table 2).

**Conclusion:**

Shorter durations of abx therapy for GNB in immunocompromised patients showed similar clinical outcomes compared to longer durations of therapy. These findings support further investigation with an appropriately powered randomized prospective trial.

**Disclosures:**

**John C. Williamson, PharmD**, Armata Pharmaceuticals: Grant/Research Support|Blue Collar Vaccines and Therapeutics: Board Member|Blue Collar Vaccines and Therapeutics: Ownership Interest|Paratek Pharmaceuticals: Grant/Research Support|ST Pharm Co, Ltd: Grant/Research Support **Ryan C. Maves, MD**, AiCuris: Grant/Research Support|Biotest: Grant/Research Support|GeoVax: Grant/Research Support|Shionogi: Advisor/Consultant|Shionogi: Honoraria|Sound Pharmaceuticals: Grant/Research Support

